# Nobiletin Induces Protective Autophagy Accompanied by ER-Stress Mediated Apoptosis in Human Gastric Cancer SNU-16 Cells

**DOI:** 10.3390/molecules21070914

**Published:** 2016-07-14

**Authors:** Jeong Yong Moon, Somi Kim Cho

**Affiliations:** 1 Subtropical Horticulture Research Institute, Jeju National University, Jeju 63243, Korea; owenmjy@jejunu.ac.kr; 2 Faculty of Biotechnology, College of Applied Life Sciences, SARI, Jeju National University, Jeju 63243, Korea

**Keywords:** apoptosis, autophagy, ER stress, GRP78, nobiletin, SNU-16

## Abstract

Nobiletin, a major component of citrus fruits, is a polymethoxyflavone derivative that exhibits anticancer activity against several forms of cancer, including SNU-16 human gastric cancer cells. To explore the nobiletin-induced cell death mechanism, we examined the changes in protein expression caused by nobiletin in human gastric cancer SNU-16 cells by means of two-dimensional gel electrophoresis (2-DGE), followed by peptide mass fingerprinting (PMF) analysis. Seventeen of 20 selected protein spots were successfully identified, including nine upregulated and eight downregulated proteins. In nobiletin-treated SNU-16 cells the glucose-regulated protein 78 kDa (GRP78) mRNA level was induced most significantly among six proteins related to cell survival and death. Western blot analysis was used to confirm the expression of GRP78 protein. We detected increases in the levels of the ER-stress related proteins inositol requiring enzyme 1 alpha (IRE1-α), activating transcription factor 4 (ATF-4), and C/EBP homology protein (CHOP), as well as GRP78, in response to nobiletin in SNU-16 cells. Furthermore, the ER stress-mediated apoptotic protein caspase-4 was proteolytically activated by nobiletin. Pretreatment with chloroquine, an autophagy inhibitor, strongly augmented apoptosis in SNU-16 cells, as evidenced by decreased cell viability, an increased number of sub-G1 phase cells and increased levels of cleaved PARP. Our results suggest that nobiletin-induced apoptosis in SNU-16 cells is mediated by pathways involving intracellular ER stress-mediated protective autophagy. Thus, the combination of nobiletin and an autophagy inhibitor could be a promising treatment for gastric cancer patients.

## 1. Introduction

Nobiletin (5,6,7,8,3′,4′-hexamethoxyflavone) is a citrus fruit-derived polymethoxyflavonoid [1] that suppresses matrix metalloproteinase (MMP)-7 expression, presumably by blocking AP-1 activity [2]. Nobiletin can directly inhibit mitogen-activated protein/extracellular signal-regulated kinase kinase (MEK) activity and decrease the sequential phosphorylation of extracellular signal-regulated kinases (ERK), leading to antitumor activity by suppressing MMP expression in HT-1080 cells [3]. We previously demonstrated that nobiletin induced apoptosis and had synergistic anticancer effects with 5-fluorouracil in p53-mutated SNU-16 cells [4]. Despite these findings, the target proteins in nobiletin-treated cancer cells have not been identified and the mechanisms underlying the anticancer properties of nobiletin remain unclear. To facilitate more effective therapeutic approaches, we used two-dimensional gel electrophoresis (2-DGE) and peptide mass fingerprinting (PMF) proteomic analysis to identify proteins involved in the anticancer mechanisms of nobiletin using the human gastric cell line SNU-16.

The endoplasmic reticulum (ER) performs several functions, including protein folding and transport, and regulation of the intracellular calcium concentration. Cells trigger the unfolded protein response (UPR) as a self-protective mechanism upon the disruption of ER functions via the accumulation of unfolded/misfolded proteins in the ER [5]. Under normal conditions, the ER stress sensors inositol-requiring-1α (IRE1-α), pancreatic ER kinase or PKR-like ER kinase (PERK), and activating transcription factor 6 (ATF6) bind to immunoglobulin heavy chain binding protein (BiP)/GRP78. These sensors are released from BiP/GRP78 under conditions of ER stress and transfer downstream signals to the cytoplasm. IRE1-α, a transmembrane protein in the endoplasmic reticulum (ER), which functions as a sensor and transducer of ER stress, activates X-box-binding protein-1 (XBP-1) and several UPR target genes. ER stress-mediated apoptosis is triggered by activation of the ER membrane resident caspase-12 (mice) and caspase-4 (humans) and the induction of C/EBP homology protein (CHOP) [6]. ER stress-induced apoptotic signaling has been studied to identify potential targets for therapeutic intervention in diseases associated with ER stress [7]. The CHOP-mediated downregulation of Bcl-2 can favorably influence pro-apoptotic Bcl-2 family proteins and cell death [8,9].

Autophagy is a cellular defense mechanism involving the degradation and recycling of cytoplasmic constituents. Several cell signaling pathways regulate autophagy, including those involving phosphatidyl inositol 3-kinase (PI3K)/Akt/mammalian target of rapamycin (mTOR). Studies have shown that the inhibition of Akt and its downstream target mTOR contribute to autophagy initiation [10]. During autophagy, autophagosomes engulf cytoplasmic components, including cytosolic proteins and organelles. Concomitantly, a cytosolic form of LC3 (LC3-I) is conjugated to phosphatidylethanolamine to form LC3-phosphatidylethanolamine conjugate (LC3-II), which is recruited to autophagosomal membranes [11].

In the present study, we examined the changes in protein expression caused by nobiletin in human gastric cancer SNU-16 cells using 2-DGE and proteomic analysis to explore the mechanism of nobiletin-induced cell death. The ER-stress-related protein GRP78 was identified as a potential target for nobiletin, and our results demonstrate that nobiletin induces ER stress-mediated apoptosis in SNU-16 cells. Moreover, protective autophagy was followed by apoptosis. Thus, we propose that combined treatment with nobiletin and an autophagy inhibitor could be a promising treatment for gastric cancer patients.

## 2. Results

### 2.1. 2-DGE and Protein Identification by MS

**Figure 1:**
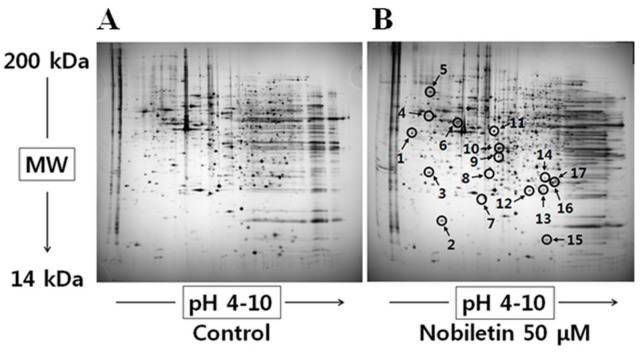
Representative protein maps from human gastric cancer SNU-16 cells that were treated with (**A**) DMSO or (**B**) 50 μM nobiletin for 24 h. Spots with arrows represent proteins that changed their expression after nobiletin treatment; the PMF-based identification of these spots is summarized in Table 1.

**Figure 2:**
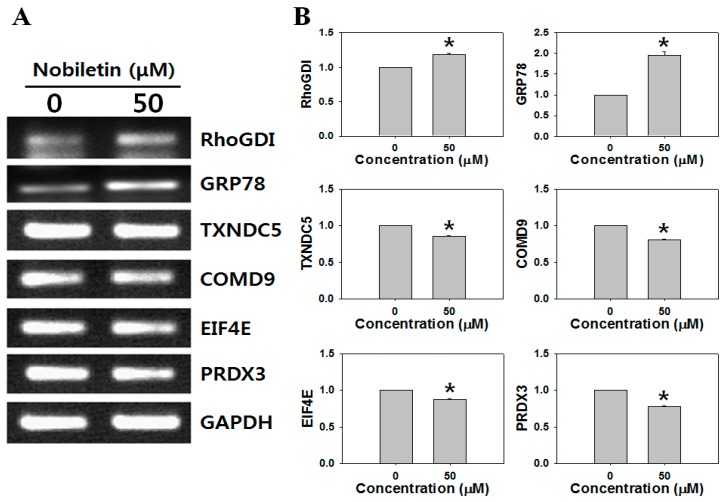
Effect of nobiletin on mRNA levels in SNU-16 cells. (**A**) Expression levels of six genes related to cell survival and death were determined by RT-PCR in SNU-16 cells treated with or without 50 μM nobiletin for 24 h; (**B**) The intensities of RT-PCR bands were quantified using ImageJ software. * *p* < 0.01.

Quantitative and qualitative analyses were performed twice, and the change in expression was considered significant if the intensity of the corresponding spot was reproducibly different and increased or decreased in relative volume between the cells treated with 50 μM nobiletin and DMSO (negative control) for 24 h. The treatment time was chosen based on the finding that 24 h-treated SNU-16 cells had 65.30% ± 9.76% viability (data not shown) compared to 41.65% viability of the cell treated cells for 48 h [4]. Sixty-two different spots between nobiletin treatment and the negative control were identified by repeated 2-DGE analysis. We excluded spots that were too weak and selected 20 spots that were dense on both gels. Seventeen of 20 selected protein spots were successfully identified (Figure 1), including nine upregulated and eight downregulated proteins (Table 1). mRNA levels of the six proteins related to cell survival and death—including the Rho GDP dissociation inhibitor 1 (RhoGDI), glucose-regulated protein 78 kDa (GRP78), thioredoxin domain-containing protein 5 (TXNDC5), COMM domain-containing protein 9 (COMD9), eukaryotic translation initiation factor 4E (EIF4E), and peroxiredoxin 3 (PRDX3)—were analyzed by RT-PCR. Among these, the GRP78 mRNA level was most significantly induced in nobiletin-treated SNU-16 cells (Figure 2). Next, the changes in GRP78 levels in SNU-16 cells were confirmed by western blotting.

### 2.2. Nobiletin Induced ER Stress-Mediated Apoptosis

GRP78 regulates a variety of biological functions, including protein folding, the activation of transmembrane ER stress sensors, and cell survival [12]. Importantly, GRP78 signaling is crucial for cell survival/apoptosis via various signaling pathways [13,14]. In addition, previous reports found that nobiletin associated with ER stress by upregulated expression of DDIT3 (DNA-damage-inducible transcript 3, also known as CHOP) and TRIB3 (tribbles homolog 3 protein, also known as TRB3) genes and proteins, which are well known to contribute to apoptosis caused by ER stress, in SK-N-SH human neuroblastoma, HuH-7 human hepatoma, and 3Y1 rat fibroblast cell lines [15,16,17]. Therefore, we investigated whether ER stress was involved in response to nobiletin treatment in SNU-16 cells. XBP-1 mRNA is constitutively expressed at a low level as an intron-containing precursor mRNA, unspliced XBP-1 (XBP-1u), which is subject to an inositol-requiring-1 α (IRE1-α)-mediated splicing reaction upon ER stress to produce the active form of XBP-1 mRNA, a spliced form of XBP-1 (XBP-1s) [18].

**Figure 3:**
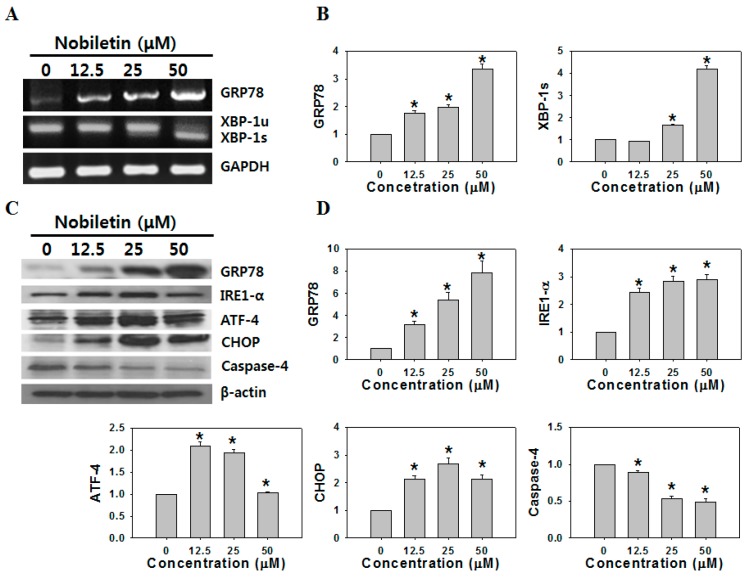
Effect of nobiletin on the expression of ER stress-related genes and proteins. (**A**) GRP78 expression and the splicing of endogenous XBP1 (XBP1u, unspliced XBP1; XBP1s, spliced XBP1) was examined by RT-PCR in SNU-16 cells treated with various concentrations of nobiletin for 24 h; (**B**) The intensities of RT-PCR bands were quantified using the ImageJ software; (**C**) ER stress- related protein expression was analyzed by western blotting in SNU-16 cells treated with various concentrations of nobiletin for 24 h; (**D**) The intensities of western blot bands were quantified using the ImageJ software. Data represent the means ± SD of at least three independent experiments. * Compared with the vehicle group, *p* < 0.01.

Excision of a 26-nucleotide-long intron creates XBP-1s, resulting in expression of an active and stable transcription factor that regulates transcription of target genes involved in protein folding and ER-associated degradation [19]. As shown in Figure 3A,B, the level of the spliced form of XBP-1 (XBP-1s) was significantly increased by nobiletin treatment, suggesting that ER stress is involved in the response to nobiletin. Next, we determined whether ER stress-mediated apoptosis is induced in response to nobiletin treatment. As shown in Figure 3C,D, the levels of the ER-stress markers inositol-requiring-1 α (IRE1-α), activating transcription factor 4 (ATF-4), and C/EBP homology protein (CHOP) were increased, and the level of inactive procaspase-4 was decreased. The cleaved form of caspase-4 was not detected even though we have tried western blot of caspase-4 many times. We speculate that this may be due to the low level of caspase-4 in the cell since Tatsuta et al. [20] also reported the degradation of procaspase-4 as a sign of namely activation of caspase-4. As proteolytic cleavage of caspase-4 and upregulation of CHOP are reportedly involved in ER stress-mediated apoptosis [6], these results suggest that nobiletin induces ER stress-mediated apoptosis in SNU-16 cells.

### 2.3. Nobiletin Induced Autophagy in SNU-16 Cells

**Figure 4:**
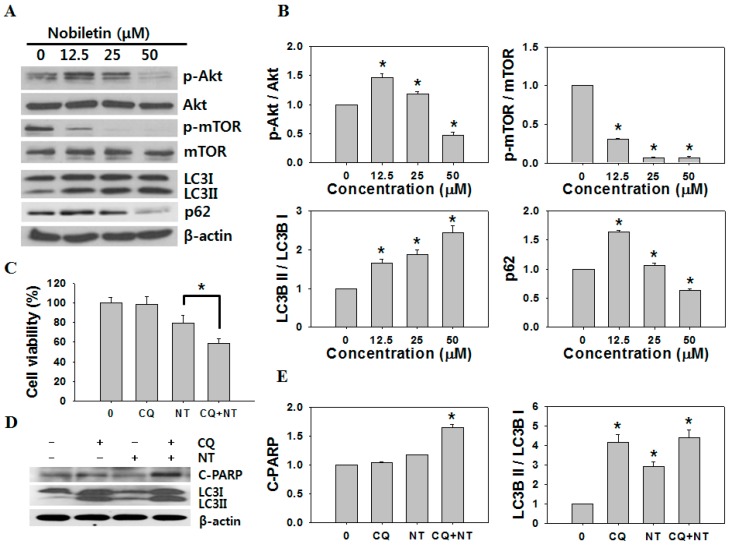
Autophagy induction due to nobiletin and inhibition of autophagy enhance the anticancer activity of nobiletin. (**A**) Western blotting for Akt, p-Akt, mTOR, p-mTOR, LC3, p62, and β-actin after treatment of cells with the indicated concentrations of nobiletin for 24 h; (**B**) The intensities of western blot bands were quantified using ImageJ software. * *p* < 0.01; (**C**) Cell viability (MTT) assay and (**D**) western blotting were performed after pretreatment with (+) or without (−) 40 μM chloroquine (CQ) for 2 h followed by treatment with 25 μM nobiletin (NT) for 24 h; (**E**) The intensities of western blot bands were quantified using ImageJ software. * *p* < 0.01.

Recent studies show that autophagy plays key roles in cancer treatment and is associated with apoptosis [21]. Furthermore, numerous chemotherapeutic drugs have been found to induce cellular autophagy [22,23]. To test whether nobiletin-induced apoptosis can induce autophagy, we examined the levels of Akt/mTOR signaling proteins, either in phosphorylated (activated) or unphosphorylated forms, by western blotting. PI3K/Akt and the downstream mTOR play important roles in regulating cell proliferation, cell cycle, and are key regulators of autophagy initiation [24]. Nobiletin treatment caused a significant decrease in phosphorylated Akt and mTOR, and it increased the ratio of LC3B II/LC3B I and decreased the level of p62, indicating that p62 is degraded by autophagy through a direct interaction with LC3 (Figure 4A,B).

### 2.4. Inhibition of Autophagy Increases Nobiletin-Induced Apoptosis

Autophagy may have a protective effect on tumor cells and therapy-induced cell death can be potentiated through autophagy inhibition [25]; thus, we determined whether the autophagy signal induced by nobiletin was pro-survival or pro-death. Cells were treated with chloroquine (CQ), which inhibits the fusion of autophagosomes and lysosomes, for 2 h before nobiletin (NT) treatment. As shown in Figure 4C, the proliferation of NT-treated SNU-16 cells was significantly reduced when cells were pre-treated with CQ, while CQ treatment alone did not affect cell viability. Western blotting revealed that NT increased cleaved PARP in the presence of CQ (Figure 4D,E). We also examined the sub-G1 population in SNU-16 cells pretreated with CQ followed by nobiletin treatment. When cells were treated with nobiletin alone for 24 h, 17.2% ± 2.9% of the cells were in sub-G1 phase (Table 2). In cells pretreated with CQ and then treated with nobiletin, the sub-G1 population increased to 23.0% ± 3.1%. These findings indicate that nobiletin-induced autophagy plays a protective role against apoptosis and that the inhibition of nobiletin-induced autophagy could enhance apoptosis in SNU-16 cells.

## 3. Discussion

We identified the cellular effectors of nobiletin using 2-DGE, which may provide insight into the intracellular cell death signaling and underlying mechanism exerted by nobiletin. Our proteomic screen revealed dramatic differences between the nobiletin-treated cells and controls (Table 1). We hypothesized that proteins related to cell proliferation and survival would be identified since we previously reported that nobiletin induced apoptosis in SNU-16 cells [4]. We identified 17 proteins, including nine upregulated and eight downregulated proteins. Among these proteins, six are reportedly related to cell survival and death; i.e., RhoGDI, GRP78, TXNDC5, COMD9, EIF4E, and PRDX3, which reportedly affect Rho GTPase activity, ER stress, cancer progression, NF-κB suppression, cell growth, cell cycle progression, and apoptosis [26,27,28,29,30,31]. RT-PCR revealed that GRP78 was the most strongly induced of all of the nobiletin-responsive proteins (Figure 2). GRP78, or BiP, is a major ER-localized chaperone that is conserved from yeast to humans [32,33]. GRP78 is highly expressed under stressful conditions, including low glucose, low oxygen, and low calcium, and it is responsible for maintaining ER stability and cell protection [34]. GRP78 is closely associated with carcinogenesis and tumor development, differentiation, and drug resistance [35]. The significant increase in GRP78 caused by nobiletin suggests the involvement of ER stress in the mechanism of SNU-16 cell death. As shown in Figure 3, IRE1-α, ATF-4, CHOP, and caspase-4, which are responsible for UPR signaling, were significantly activated.

The ER plays an important role in protein folding, assembly, modification, and transport. UPRs can occur in most tumor cells, and they can promote tumor cell growth and induce GRP78 expression in the ER [36]. The central feature of this adaptive response was suggested to be maintenance of the expression of proteins that facilitate cell survival (e.g., GRP78). In addition, PI3K/Akt is a critical mediator of growth factor-induced cell survival and can suppress cell death induced by a variety of apoptotic stimuli [37]. Inhibition of the Akt/mTOR pathway is correlated with autophagy induction [38,39], and ER stress-induced autophagy is responsible for the effective removal of toxic proteins from the ER and cell protection [40].

However, as indicated in Figure 4A,B, the p-Akt level was increased by application of 12.5 µM nobiletin. This is reminiscent of recent studies that nobiletin can enhance the circadian clock which is known to regulate insulin signaling, specifically AKT activation [41,42]. Nobiletin potently protects against metabolic syndrome in a clock-dependent manner and it remodels circadian and metabolic gene expression [43]. On the other hand, a negative feedback loop has been described whereby mTOR/S6K1 activation attenuates PI3K signaling by suppressing insulin receptor substrate-1 (IRS1) function, a mediator of insulin receptor–dependent activation of PI3K [44]. It has been proposed that mTORC1 inhibition relieves inhibition of the PI3K pathway thorough inactivation of S6K1, thereby activating Akt in low concentration of nobiletin [45].

During the initial stages of autophagy, cellular proteins, organelles, and the cytoplasm are sequestered and engulfed by intracellular double-membrane-bound structures called autophagosomes. In turn, autophagosomes fuse with lysosomes, and their contents are degraded by lysosomal hydrolases [46]. Because p62 binds to ubiquitin and to LC3, it is both a selective autophagy substrate and a cargo receptor for autophagic degradation of ubiquitinated targets [47]. p62 forms cytosolic inclusion bodies distinct from aggresomes, which contain ubiquitinated protein aggregates that are subsequently degraded by autophagy [48]. Therefore, p62 is considered to act as a receptor for ubiquitinated proteins, which it sequesters into the autophagosome, and impaired autophagy is accompanied by accumulation of p62. Also, p62 is a stress response protein that is strongly induced at the mRNA and protein levels by exposure to oxidants, sodium arsenite, cadmium, ionophores, and proteasome inhibitors [49]. Our results demonstrate an immediate increase in the level of p62 during the initial stages of autophagy (12.5 μM nobiletin) followed by a gradual decrease in a dose-dependent manner, demonstrating that eventually autophagy is accompanied by degradation of p62 following application of nobiletin. Therefore, the combination of nobiletin with an autophagy inhibitor may improve the therapeutic outcome in gastric cancer.

## 4. Materials and Methods

### 4.1. Chemicals and Reagents

Nobiletin, propidium iodide (PI), and anti-β-actin antibodies were purchased from Sigma-Aldrich (St. Louis, MO, USA). RPMI-1640 medium, trypsin/EDTA, fetal bovine serum (FBS), penicillin, streptomycin, goat anti-rabbit IgG, and goat anti-mouse IgG secondary antibodies were obtained from Life Technologies (Carlsbad, CA, USA). Dimethyl sulfoxide (DMSO) and MTT were purchased from Amresco (Solon, OH, USA). Antibodies against various proteins for Western blotting such as cleaved PARP, caspase-4, IRE1-α, ATF-4, CHOP, p-Akt, Akt, p-mTOR, mTOR, and LC3B were obtained from Cell Signaling Technology (Danvers, MA, USA). Anti-GRP78 antibodies were purchased from Santa Cruz Biotechnology (Santa Cruz, CA, USA).

### 4.2. Cell Culture

SNU-16 cells were maintained at 37 °C in a humidified atmosphere containing 5% CO_2_ in RPMI-1640 medium containing 10% heat-inactivated FBS, 100 U/mL penicillin, and 100 μg/mL streptomycin. Exponentially growing cells were treated with various concentrations of the solvent fractions as indicated.

### 4.3. 2-DGE Reagents

Urea, thiourea, 3-[(3-cholamidopropy) dimethyammonio]-1-propanesulfonate (CHAPS), dithio-threitol (DTT), benzamidine, Bradford solution, acrylamide, iodoacetamide, bisacrylamide, SDS, acetonitrile, trifluoroacetic acid, and α-cyano-4-hydroxycinnamic acid were purchased from Sigma-Aldrich (electrophoresis grade, ACS reagents, Ultrapure). Pharmalyte (pH 3.5–10) was obtained from Amersham Biosciences (Piscataway, NJ, USA) and IPG DryStrips (pH 4–10 NL, 24 cm) were obtained from Genomine (Gyeongsangbuk-do, Korea). Modified porcine trypsin (sequencing grade) was purchased from Promega (Madison, WI, USA).

### 4.4. 2-DGE

IPG strips were equilibrated for 12–16 h with 7 M urea, 2 M thiourea containing 2% CHAPS, 1% DTT, and 1% Pharmalyte, and loaded with 200 μg of sample. Isoelectric focusing (IEF) was performed at 20 °C using a Multiphor II electrophoresis unit and EPS 3500 XL power supply (Amersham Biosciences) following the manufacturer’s instructions. For IEF, the voltage was linearly increased from 150 to 3500 V over 3 h for sample entry followed by a constant voltage (3500 V) with focusing complete after 96 kVh. Prior to the second dimension, the strips were incubated for 10 min in equilibration buffer (50 mM Tris-HCl, pH 6.8, containing 6 M urea, 2% SDS, and 30% glycerol) first with 1% DTT and then with 2.5% iodoacetamide. Equilibrated strips were inserted onto SDS-polyacrylamide gels (20 × 24 cm, 10%–16%). SDS-PAGE was performed using the Hoefer DALT 2D system (Amersham Biosciences) following the manufacturer’s instructions. The two-dimensional gels were run at 20 °C for 1700 Vh then silver-stained as described, except that the fixation and sensitization steps with glutaraldehyde were omitted [50].

### 4.5. Image Analysis

A quantitative analysis of digitized images was performed using PDQuest (version 7.0; Bio-Rad, Hercules, CA, USA) software according to the manufacturer’s protocols. The quantity of each spot was normalized by the total valid spot intensity. Protein spots were selected based on significant expression variation deviating more than twofold in expression level compared with control or normal samples.

### 4.6. Peptide Mass Fingerprinting (PMF)

For protein identification using PMF, protein spots were excised, digested with trypsin (Promega, Madison, WI, USA), mixed with α-cyano-4-hydroxycinnamic acid in 50% acetonitrile/0.1% TFA, and subjected to matrix-assisted laser desorption/ionization- time-of-flight (MALDI-TOF) analysis (Ettan MALDI-TOF Pro; Amersham Biosciences) as described previously [51]. Spectra were collected from 350 shots per spectrum over the 600–3000 *m*/*z* range and calibrated by two-point internal calibration using trypsin autodigestion peaks (*m*/*z* 842.5099 and 2211.1046). The peak list was generated using the Ettan MALDI-TOF Pro Evaluation Module (version 2.0.16; GE Healthcare, Little Chalfont, UK). The thresholds used for peak-picking were 5000 for minimum resolution of monoisotopic masses and 2.5 for S/N. The search program MASCOT, developed by Matrix Science (Boston, MA, USA), was used for protein identification. The following parameters were used for the database search: trypsin as the cleaving enzyme, a maximum of one missed cleavage, iodoacetamide (Cys) as a complete modification, oxidation (Met) as a partial modification, monoisotopic masses, and a mass tolerance of ± 0.1 Da. Probability scoring was used as PMF acceptance criteria.

### 4.7. Reverse Transcription-Polymerase Chain Reaction (RT-PCR)

Total RNA was extracted using TRIzol reagent (Invitrogen, Frederick, MO, USA) and precipitated in ethanol. cDNA synthesis was performed with 1 μg of total RNA by reverse transcription (Promega). The primers used were: Rho GDP dissociation inhibitor 1 (RhoGDI) [5′-GAGCCTGCGAAAGTACAAGG-3′ (sense), and 5′-TCCTTCAGCACAAACGACTG-3′ (antisense)]; glucose-regulated protein 78 kDa (GRP78) [5′-CTCCTGAAGGGGAACGTCTG-3′ (sense), and 5′-AACACTTTCTGGACGGGCTT-3′ (antisense)]; thioredoxin domain-containing protein 5 (TXNDC5) [5′-GCACAAGGCGACCACTTTAT-3′ (sense), and 5′-ATCCCGCTTTCCCTTGTACT-3′ (antisense)]; COMM domain-containing protein 9 (COMD9) [5′-CCACCAAAACCTCAAAAACC-3′ (sense), and 5′-AGGCTGGGATCTTCTTGGAT-3′ (antisense)]; eukaryotic translation initiation factor 4E (EIF4E) [5′-CAGATGGGCACTCTGGTTTT-3′ (sense), and 5’-CTCCCCGTTTGTTTTTCTCA-3′ (antisense)]; peroxiredoxin 3 (PRDX3) [5′-GTTGTCGCAGTCTCAGTGGA-3′ (sense), and 5′-GACGCT CAAATGCTTGATGA-3′ (antisense)]; XBP-1 [5′-TTACGAGAGAAAACTCATGGCC-3′ (sense), and 5′-GGGTCCAAGTTGTCCAGAATGC-3′ (antisense)]; and GAPDH [5′-GAGAAGGCTGGGGCTC ATTT-3′ (sense), and 5′-AGTGATGGCATGGACTGTGG-3′ (antisense)]. The RT-PCR conditions were 95 °C pre-denaturation for 5 min, followed by 95 °C denaturation for 40 s, 55–60 °C annealing for 30 s, and 72 °C extension for 40 s, for a total of 35 cycles.

### 4.8. Western Blotting

Harvested cells were washed with PBS and then lysed with RIPA lysis buffer containing protease inhibitor cocktail (BioVision, Milpitas, CA, USA) and PMSF. Protein concentrations were determined using BCATM Protein Assay Reagent (Pierce, IL, USA). Equal amounts of protein were separated by 7.5%–15% SDS-PAGE and transferred to a polyvinylidene difluoride (PVDF) membrane (Merck Millipore, Darmstadt, Germany) using glycine transfer buffer (192 mM glycine, 25 mM Tris-HCl (pH 8.8), and 20% (*v*/*v*) methanol). After blocking with 5% skim milk, the membrane was incubated for 2 h with primary antibodies and then for 30 min with secondary antibodies in milk containing Tris-buffered saline and 0.1% Tween 20. The membrane was then exposed to X-ray film (AGFA, Mortsel, Belgium) and the protein bands were detected using the WEST-ZOL^®^ plus Western Blot Detection System (iNtRON, Gyeonggi-do, Korea).

### 4.9. Cell Viability Assay

Cell growth inhibition was examined using an MTT assay. SNU-16 was seeded in 96-well culture plates (5 × 10^4^ cells/mL). After incubation overnight, the cells were treated with 40 µM chloroquine (CQ) and 25 µM nobiletin (NT). Next, 0.1 mg of MTT was added to each well and the cells were incubated at 37 °C for 4 h. The medium was removed and 150 µL of DMSO was then added to each well to dissolve the formazan crystals. The absorbance at 570 nm was measured using a microplate reader (Tecan, Salzburg, Austria).

### 4.10. Flow Cytometry

For cell cycle distribution analysis, cells (5 × 10^4^ mL) were plated and left untreated or treated with samples for 24 h. The cells were then collected, fixed in 70% ethanol, washed in 2 mM EDTA in phosphate-buffered saline (PBS), resuspended in 1 mL of PBS containing 1 mg/mL RNase and 50 mg/mL PI, and incubated at 37 °C in the dark for 30 min. The DNA content was analyzed using a FACScan flow cytometer (BD Biosciences, San Jose, CA, USA). The population of cells in each cell cycle phase was determined using CellQuest software (BD Dickson, Franklin Lakes, NJ, USA). The sub-G1 population showed apoptosis-associated chromatin degradation.

### 4.11. Statistical Analyses

All experiments were repeated at least three times. The data are expressed as means ± standard deviation (SD). A statistical analysis was performed using one-way analysis of variance (ANOVA); values of * *p* < 0.01 were considered to indicate statistical significance.

## 5. Conclusions

In conclusion, using a global proteomics approach, we identified the nobiletin-regulated protein GRP78 and showed that nobiletin induced ER stress-mediated apoptosis and autophagy in SNU-16 cells via the downregulation of Akt/mTOR signaling. Collectively, nobiletin-induced ER stress plays a crucial role in anticancer activity against SNU-16 gastric cancer cells. These results may promote the development of efficacious therapies combining nobiletin and chloroquine for gastric cancer patients.

## Figures and Tables

**Table 1:** Proteins from nobiletin-treated SNU-16 cells identified by PMF spectrometry of spots excised from two-dimensional gels.

No. ^a^	Identified Protein	Score	MW ^b^ (Da)/PI ^c^	Fold Change ^d^
1	Spermine synthase	120	41698/4.87	4.5
2	Chain B, histocompatibility antigen Hla-Dm	67	21972/6.70	3.6
3	Rho GDP-dissociation inhibitor 1	135	23250/5.02	1.9
4	Mitochondrial ATP synthase, H-transporting F1 complex beta subunit	136	48083/4.95	3.2
5	78 kDa glucose-regulated protein	241	72402/5.07	6.4
6	TXNDC5 protein	233	41028/5.57	5.1
7	COMM domain-containing protein 9	91	17436/6.41	0.3
8	Eukaryotic translation initiation factor 4E	73	15290/6.73	0.4
9	Chain A, the high resolution structure of annexin Iii shows differences with annexin V	183	36480/5.63	4.2
10	Capping protein (actin filament) muscle Z-line, alpha 2	93	31898/6.46	56.1
11	EF-hand calcium binding domain 6	85	48889/9.67	2.4
12	Peroxiredoxin 3	66	11158/6.06	0.8
13	Proteasome subunit beta type-3	71	23219/6.14	0.7
14	Chain A, crystal structure of the protein disulfide isomerase-related chaperone Erp29	66	27220/7.07	0.6
15	Fatty acid-binding protein	64	15497/6.60	0.6
16	Peroxiredoxin-5	240	25133/6.00	0.6
17	Proteasome subunit alpha type-6	133	27838/6.34	0.6

^a^ No: spot number; ^b^ MW: molecular weight; ^c^ PI: isoelectric point; ^d^ Fold change calculated dividing the % (vol) from the control gel by that from the nobiletin-treated gel.

**Table 2:** The percentage of SNU-16 cells in different phases of the cell cycle after nobiletin treatment with/without CQ for 24 h.

Treatment	Phase (%)			
	Sub-G1	G1	S	G2/M
Control	7.9 ± 4.7	55.3 ± 2.4	11.6 ± 1.1	25.5 ± 5.3
CQ ^a^ alone	9.6 ± 2.8	55.1 ± 4.9	11.5 ± 2.4	24.2 ± 2.7
Nobiletin ^b^ alone	17.2 ± 2.9 *	63.0 ± 1.4	4.2 ± 0.9	15.8 ± 3.3
Nobiletin + CQ	23.0 ± 3.1 *	54.4 ± 6.1	6.2 ± 2.4	16.6 ± 3.6

The results are presented as means ± standard deviation of at least three independent experiments. * *p* < 0.01. ^a^ Chloroquine (CQ) concentration; 40 µM and ^b^ nobiletin concentration; 25 µM.
